# Menaquinone-specific turnover by *Mycobacterium tuberculosis* cytochrome *bd* is redox regulated by the Q-loop disulfide bond

**DOI:** 10.1016/j.jbc.2024.108094

**Published:** 2024-12-18

**Authors:** Tijn T. van der Velden, Kanwal Kayastha, Caspar Y.J. Waterham, Steffen Brünle, Lars J.C. Jeuken

**Affiliations:** Leiden Institute of Chemistry, Leiden University, Leiden, The Netherlands

**Keywords:** ubiquinone, demethylmenaquinone, cryo-EM, respiration, substrate specificity, disulfide regulation

## Abstract

Cytochrome *bd* from *Mycobacterium tuberculosis* (*Mtbd*) is a menaquinol oxidase that has gained interest as an antibiotic target because of its importance in survival under infectious conditions. *Mtbd* contains a characteristic disulfide bond that has been hypothesized to allow for *Mtbd* activity regulation at the enzymatic level, possibly helping *M. tuberculosis* to rapidly adapt to the hostile environment of the phagosome. Here, the role of the disulfide bond and quinone specificity have been determined by reconstitution of a minimal respiratory chain and the single-particle cryo-EM structure in the disulfide-reduced form. *Mtbd* was shown to be specific for menaquinone, while regulation by reduction of the Q-loop disulfide bond decreased oxidase activity up to 90%. Structural analysis shows that a salt bridge unique to *Mtbd* keeps the Q-loop partially structured in its disulfide-reduced form, which could facilitate the rapid activation of *Mtbd* upon exposure to reactive oxygen species. We signify *Mtbd* as the first redox sensory terminal oxidase and propose that this helps *M. tuberculosis* in the defense against reactive oxygen species encountered during infection.

Cytochrome *bd* (cyt *bd*) is a terminal oxidase found in the respiratory chain across various bacterial phyla and couples the oxidation of quinols to the reduction of molecular oxygen to water (1, 2, 3). The chemical protons released by quinol oxidation are separated from the proton uptake for oxygen reduction by the cytoplasmic membrane, generating a proton motive force required for ATP synthesis (3). Compared with other terminal oxidases, cyt *bd* is distinguished by its resistance to inhibitors, such as cyanide, high affinity for oxygen, and upregulation under microaerobic conditions (4).

Recent findings highlight the significance of cyt *bd* as a key survival factor for *Mycobacterium tuberculosis* during infection, particularly in the hostile granulomas where the bacteria reside (5). Only upon inhibition of both terminal oxidases, cyt *bcc:aa*_*3*_ and cyt *bd*, bactericidal effects are observed (6). When *M. tuberculosis* cyt *bcc*:*aa*_3_ is inhibited and cyt *bd* from *M. tuberculosis* (*Mtbd*) functions as the sole terminal oxidase, it can maintain a bacteriostatic state (7, 6) and enhances resistance against numerous antibiotics, reactive oxygen species (ROS), and other toxic compounds (8, 9, 10, 11, 12, 13). This pivotal role during infection has identified *Mtbd* as a potential antibiotic target.

Despite this therapeutic potential, most of our current understanding of *Mtbd* at the molecular level is obtained from studies performed on homologous enzymes, such as *Corynebacterium glutamicum* cyt *bd* and the two *Escherichia coli* cyt *bd* isoforms, cyt *bd*-I (*Ecbd*) and cyt *bd*-II. Since these enzymes have been shown to have distinct structural features, number of subunits, and substrate-binding pockets (14, 15, 16, 17, 18), caution is required when associating prior knowledge to *Mtbd*. An important characteristic feature of *Mtbd* is a disulfide bond constraining the N-terminal Q-loop domain near the quinone-binding pocket (14). It has been hypothesized that this disulfide bond provides a regulatory switch (14, 19) and allows *M. tuberculosis* to instantly adapt to inhibition of its electron transport chain by rerouting electrons to *Mtbd*, suggesting regulation at the enzyme level in response to hostile conditions (20, 21). Redox-sensing disulfide bonds have been found in other proteins, such as transcription factors, signaling enzymes, CO_2_ reductases, and peroxidases as a defense against oxidative stress (22, 23). However, whether such a regulatory function is conferred by the *Mtbd* Q-loop remains speculative, because its study has been hampered by the need for chemical reductants to reduce the quinone substrates in the standard activity assays for cyt *bd* oxidase. Given its significance as a potential antibiotic target, it is essential to uncover the main features of *Mtbd* enzyme kinetics.

*Ecbd*, *Mtbd*, and cyt *bd*s from other species use distinct quinone subtypes for turnover. The main bacterial quinone subtypes include menaquinone (MK), demethylmenaquinone (DMK), and ubiquinone (UQ), each differing in their quinone headgroup structure and corresponding redox potential (Fig. 1*A*) (24). In addition, these quinones contain a hydrophobic isoprenoid tail made from a distinct number of isoprene units (25). By convention, the number of isoprene units is indicated by the number (n) after the quinone, for example, MK-n. *M. tuberculosis* primarily relies on MK-9 (26), whereas *E. coli* demonstrates distinct functionalities for MK-8, DMK-8, and UQ-8 (27, 28). Moreover, *E. coli* upregulates MK-8 during microaerobic conditions, coinciding with the upregulation of *Ecbd*, highlighting the adaptability of the *E. coli* quinone pool composition based on environmental requirements (24, 29).

**Figure 1:**
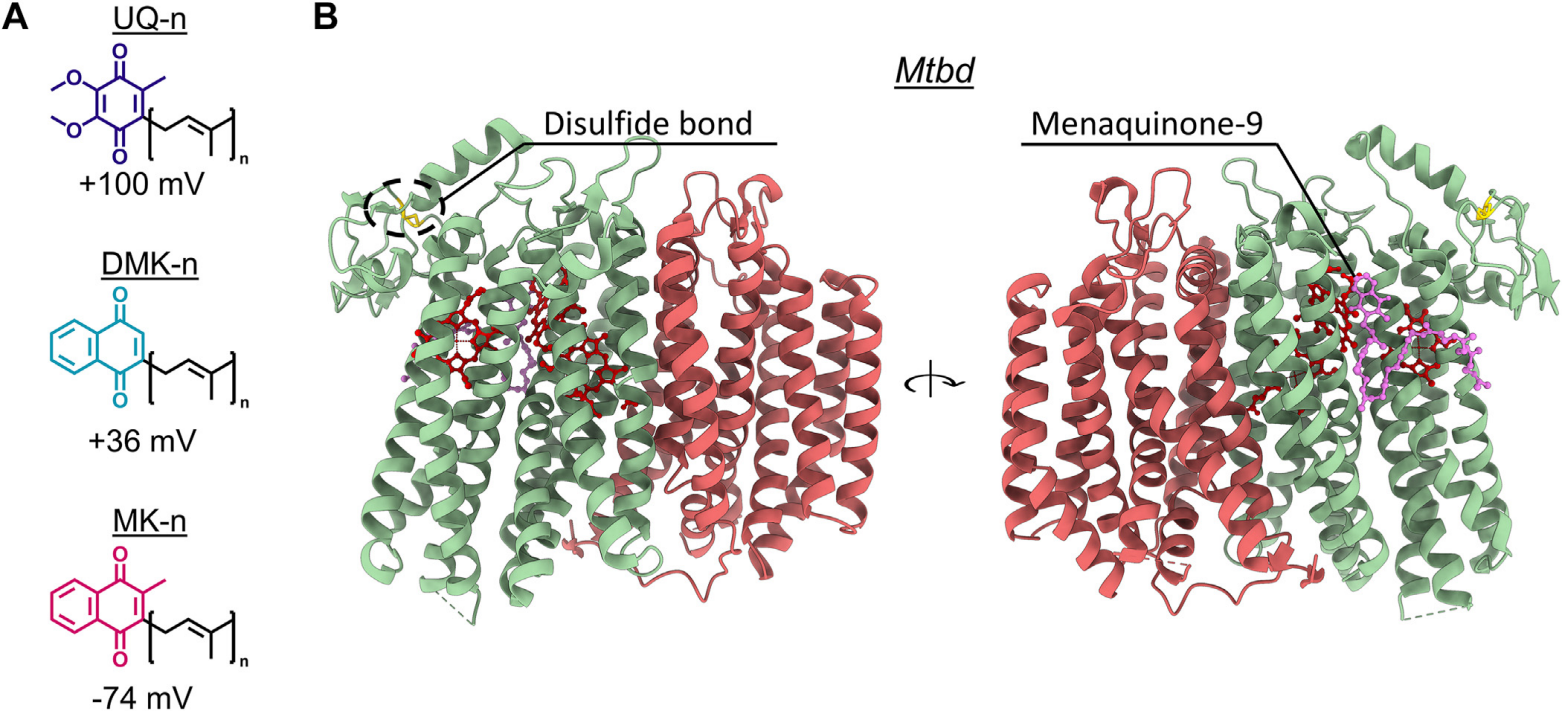
**Overview of quinone subtypes and structural features of *Mtbd.****A*, the structure of quinone analogs used in this study with the number of isoprenoid units (n) and their reduction potentials (24). *B*, oxidized structure of *Mtbd* (Protein Data Bank code: 7NKZ) (14), CydA (*green*), CydB (*red*), indicating the Q-loop disulfide bond and MK-9 (*purple*). *Mtbd*, cytochrome *bd* from *Mycobacterium tuberculosis*.

The interactions between quinones and cyt *bd* are intricate, as demonstrated by the distinct quinone-binding sites in *Ecbd* and *Mtbd* (Fig. S1). While *Ecbd* has a proposed quinone-binding pocket at the Q-loop of subunit CydA, transferring electrons to closely located heme *b*_558_ (16), *Mtbd* shows quinone binding at the back of CydA, which is hypothesized to transfer electrons directly to heme *b*_595_, bypassing the initial heme *b*_558_ (Fig. 1*B*) (14). Additional structural quinone-binding pockets are found in CydB, lacking participation in quinol oxidation (16, 18). The exact position of the *Ecbd* and *Mtbd* turnover sites remains to be determined.

Most studies make use of detergent-solubilized cyt *bd* with water-soluble quinone analogs. The use of detergent micelles, although convenient for studying cyt *bd* in solution, introduces an artificial environment that deviates from the native conditions. These detergent micelles can alter the conformation, partially unfold proteins, and strip away vital bound or annular lipids, leading to a loss of native mechanistic function and kinetic rates (30, 31). In addition, using water-soluble quinone analogs can significantly alter enzyme kinetics as the quinone isoprenoid tail has been shown to critically affect respiratory efficiency in *M. tuberculosis in vivo* (32). The evaluation of cyt *bd* quinone interactions and disulfide regulation thus requires a native membrane environment and natural quinone substrates to mimic native conditions.

To study the specific interaction of *Ecbd* and *Mtbd* with the different quinones and interrogate the role of the *Mtbd* Q-loop disulfide bond, a minimal respiratory chain was constructed in which cyt *bd* is combined with a NADH–quinone oxidoreductase, *Caldalkalibacillus thermarum* NDH-2 (Fig. 2). This approach was inspired by an approach in which complex 1 was studied using an alternative oxidase (33). Enzyme kinetics reveal that water-soluble quinone analogs lead to vast overestimations of cyt *bd* turnover rates, highlighting the need for precise experimental design. Furthermore, in contrast to *Ecbd*, *Mtbd* is subject to substrate inhibition by MK at physiologically relevant concentrations. In addition, we signify *Mtbd* as the first terminal oxidase with a redox sensory disulfide bond. Cryo-EM analysis of the disulfide-reduced *Mtbd* revealed a new interaction between Arg290 and Glu263 that might explain why *Mtbd* is readily reactivated upon reformation of the disulfide bond, for instance by exposure to H_2_O_2_. We propose that redox regulation enables *Mtbd* to quickly adapt its activity to environmental redox pressures in defense against ROS encountered by *M. tuberculosis* during infection.

**Figure 2:**
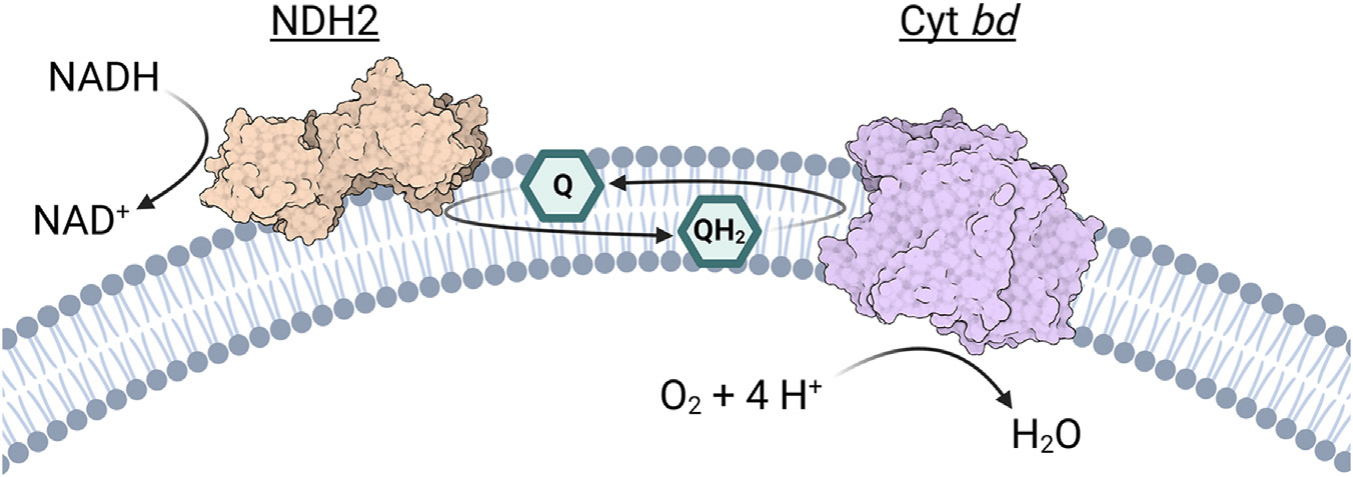
**A minimal respiratory chain proteoliposomal system to interrogate specific interactions between quinones and cyt *bd*.** This proteoliposomal system can also be used in detergent. Cyt *bd*, cytochrome *bd*.

## Results

### Cyt *bd* oxygen consumption kinetics with water-soluble quinone analogs

**Figure 3:**
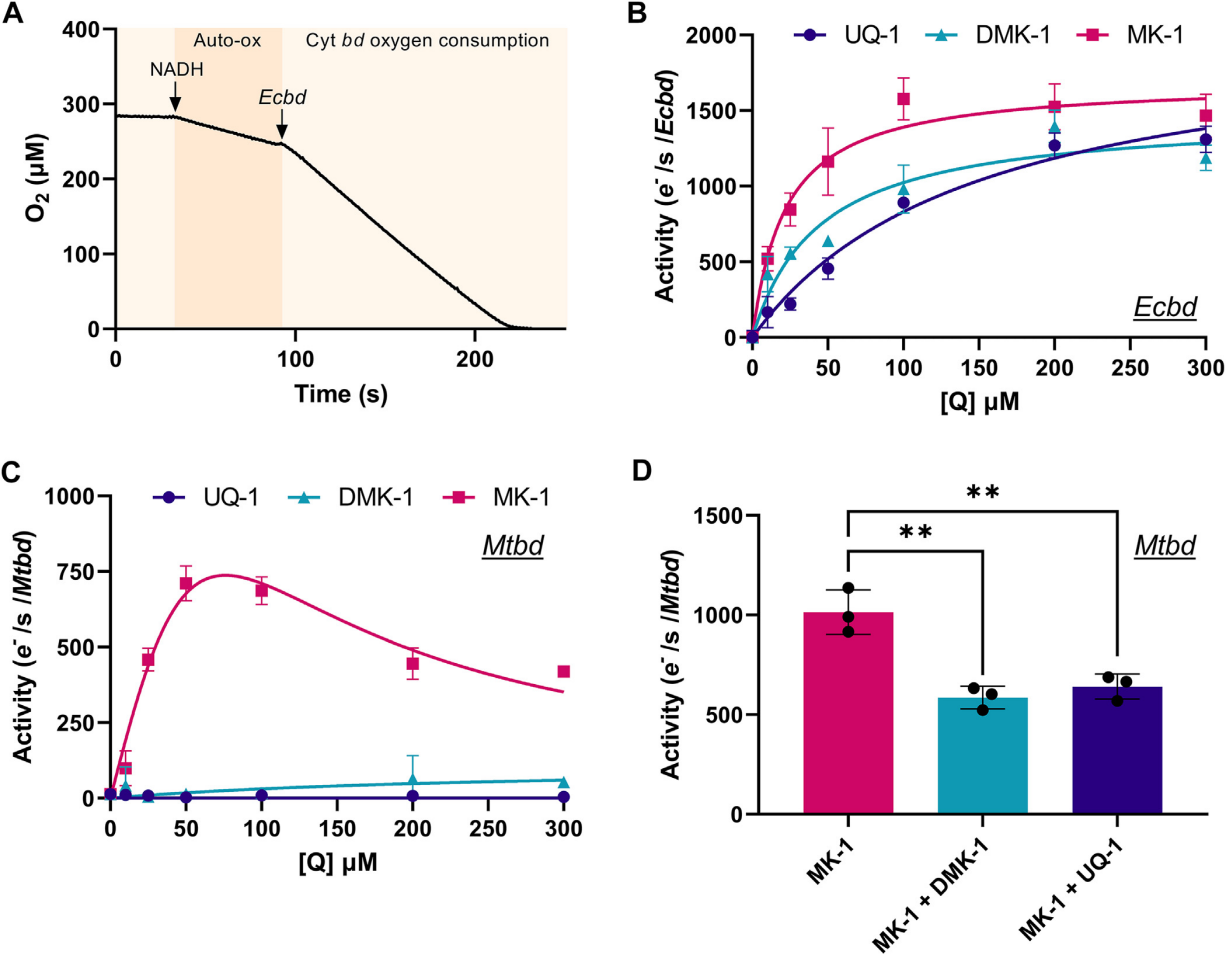
**Oxygen consumption kinetics of detergent-solubilized cyt *bd* with different quinone substrates.***A*, example of an oxygen consumption trace with MK-1 (50 μM) auto-oxidation (Auto-ox) and *Ecbd* oxygen consumption indicated. *B*, *Ecbd* and, (*C*) *Mtbd* oxygen consumption kinetics using the assay described in the text at 20 °C. *Lines* represent Michaelis–Menten fits without (*Ecbd*, see Table 1 for parameter values) or with (*Mtbd*) substrate inhibition. *D*, *Mtbd* activity with 50 μM MK-1 in the absence or presence of either DMK-1 or UQ-1 (both 300 μM). Data are represented as the average and standard deviation of triplicate measurements of two different protein preparations (n = 3). Significance is tested using an ANOVA with a Dunnett *post hoc* test in GraphPad Prism. ∗∗*p* < 0.01. Cyt *bd*, cytochrome *bd*; DMK, demethylmenaquinone; *Ecbd*, cytochrome *bd* from *Escherichia coli*; MK, menaquinone; *Mtbd*, cytochrome *bd* from *Mycobacterium tuberculosis*; UQ-1, ubiquinone 1.

*Ecbd*, *Mtbd*, and NDH-2 were expressed and purified *via* affinity and size-exclusion chromatography (SEC; Fig. S2). A minimal respiratory chain was constructed with an excess of NADH and NDH-2, such that the quinone pool remains fully reduced. Control studies with varying amounts of NDH2 confirmed that cyt *bd* activity was limiting. The enzyme activity of *Ecbd* and *Mtbd*, comparing different quinone substrates, was determined by monitoring oxygen consumption in a Clark electrode. The activity was first measured in detergent conditions using water-soluble quinone analogs. Previous reports indicate that quinols, especially menaquinols, auto-oxidize under aerobic conditions in a concentration-dependent manner (34). To account and correct for the quinol (Q) auto-oxidation rate, the oxygen consumption was quantified for each assay before the addition of cyt *bd* (Fig. 3*A*). *Ecbd* displayed standard Michaelis–Menten kinetics with a sixfold lower *K*_m_ for MK-1 compared with UQ-1 (Fig. 3*B* and Table 1). *K*_cat_ values for the three quinone analogs were similar (Table 1) ranging from 736 to 1033 Q/s (Table 1).

**Table 1:** Kinetic parameters for the oxidation of UQ-1, DMK-1, and MK-1 by *Ecbd*^a^

*K*_cat_ (Q s^−1^)			*K*_m_ (μM)			*K*_cat_/*K*_m_ (×10^−6^ M^−1^ s^−1^)		
UQ-1	DMK-1	MK-1	UQ-1	DMK-1	MK-1	UQ-1	DMK-1	MK-1
1033 ± 85	736 ± 51	846 ± 38	149 ± 26	44 ± 10	22 ± 4	6.9 ± 1.3	16.7 ± 4.0	38.5 ± 7.2

a Oxygen consumption measurements were performed in 50 mM Mops, 150 mM NaCl, 0.005% LMNG, pH 7.0 (n = 3). Data represent best Michaelis–Menten fit values with the SEM. *K_cat_* values are represented in quinones per second (2 e-/Q). *K_cat_*: UQ-1 *versus* DMK-1 (p<0.05). *K_m_* UQ-1 *versus* DMK-1 (p<0.001), UQ-1 *versus* MK-1 (p < 0.0001), DMK-1 *versus* MK-1 (p < 0.05). *K_cat_/K_m_*, errors represent the propagated SEM.

The kinetic profile of *Mtbd* was profoundly different than that of *Ecbd* (Fig. 3*C*). *Mtbd* exclusively turns over MK-1 and is subject to substrate inhibition. While substrate inhibition has been observed in other oxidases (18, 35, 36), its molecular mechanism remains undefined. Multivariate fits of the observed substrate inhibition profile indicated a large interdependency of parameters (*K*_m_; *V*_max_; inhibition constant, *K*_i_) preventing the determination of the kinetic parameters for MK-1 oxidation in spite of the good fit (Fig. 3*C*). Importantly, *Mtbd* failed to initiate turnover with either DMK-1 or UQ-1. To investigate whether this inactivity resulted from a lack of substrate binding or a thermodynamic barrier limiting quinol oxidation, the effect of UQ-1 and DMK-1 on *Mtbd* activity was assessed. It was reasoned that DMK-1 and UQ-1 will present as competitive inhibitors if they bind to the same active-site pocket as MK-1. Upon addition of an excess DMK-1 or UQ-1, oxygen consumption activity was indeed significantly inhibited, suggesting that DMK-1 and UQ-1 bind to the active site but are unable to be oxidized by *Mtbd* (Fig. 3*D*). This inhibitory effect of DMK-1 was shown to be concentration dependent and indicates a lower overall affinity for DMK-1 than MK-1 (Fig. S3).

### *Ecbd* and *Mtbd* oxygen consumption in proteoliposomes

To determine whether the observed substrate specificities in detergent are indicative of native conditions, the same principles were applied to a proteoliposomal system (Fig. 2) where cyt *bd* can be studied in a native-like lipid environment using the long isoprenoid quinones MK-9 and UQ-10.

**Figure 4:**
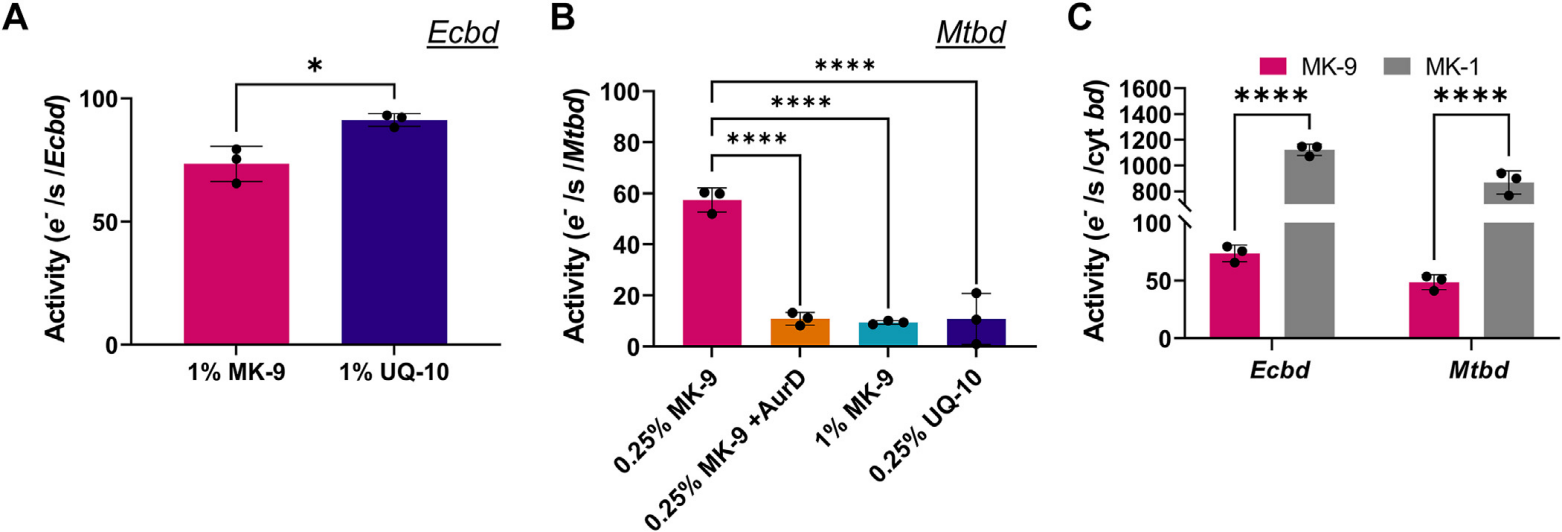
**Quinone substrate specificity of *Ecbd* and *Mtbd* in a native lipid environment.***A*, catalytic rates of *Ecbd* (20 nM) proteoliposomes containing 1% (molar; quinone/lipid) of either UQ-10 or MK-9. *B*, catalytic rates of *Mtbd* (20 nM) with different concentrations of either UQ-10 or MK-9 and inhibited by AurD. *C*, reconstituted *Ecbd* and *Mtbd* activity with native MK-9 (*Ecbd* 1% [molar; quinone/lipid] MK-9, *Mtbd* 0.25% [molar; quinone/lipid] MK-9) *versus* MK-1 analog (50 μM). Data are represented as the average and standard deviation from three individual proteoliposome reconstitutions. Significance is tested using an ANOVA with a Dunnett *post hoc* test in GraphPad Prism. ∗*p* < 0.05, ∗∗∗∗*p* < 0.0001. *Ecbd*, cytochrome *bd* from *Escherichia coli*; MK-9, menaquinone 9; *Mtbd*, cytochrome *bd* from *Mycobacterium tuberculosis*; UQ-10, ubiquinone 10.

*Mtbd* and *Ecbd* were reconstituted in liposomes containing 0.25%–1% (molar; quinone/lipid) of the desired quinone (MK-9 or UQ-10) to achieve maximum catalytic rates. The *Mtbd* or *Ecbd* concentration after reconstitution was quantified using the Soret band of a detergent-solubilized sample. Native quinone concentrations in bacterial membranes are estimated between 0.3 and 1.5% (see Supporting information). Furthermore, 1% MK-9 or UQ-10 approximately coincides with a membrane concentration in the order of 10 to 15 mM (see Supporting information, well above the *K*_m_ determined for *Ecbd*. We note however, that for the water-soluble quinone analogs, an unknown equilibrium exists between quinones in solution and quinones in detergent micelles, making the two systems difficult to directly compare. Consistent with the UQ-1–MK-1 kinetics, *Ecbd* showed a marginally higher catalytic rate with UQ-10 than MK-9 (Fig. 4*A*). In addition, *Mtbd* displayed the same specificity for MK turnover, confirming the quinone analog data (Fig. 4*B*). Substrate inhibition for *Mtbd* was confirmed by increasing the MK concentration from 0.25% to 1%, resulting in a significant decrease in enzyme activity.

To study the effect of the quinone isoprenoid chain on cyt *bd* activity, *Ecbd* and *Mtbd* were reconstituted in proteoliposomes, and turnover was measured with either membrane-embedded MK-9 or water-soluble MK-1. Surprisingly, the isoprenoid chain length of the quinone was shown to have a significant effect on the maximum catalytic rate of cyt *bd* (Fig. 4*C*). Utilizing the natural membranous quinones resulted in 10-fold lower catalytic rates, emphasizing the role of the isoprenoid tail in substrate binding, *via* either diffusion or binding kinetics. This highlights that previously reported catalytic rates using quinone analogs can substantially overestimate the catalytic rates achieved *in vivo* (16, 18, 31).

### *Mtbd* activity is regulated by a unique Q-loop disulfide bond

**Figure 5:**
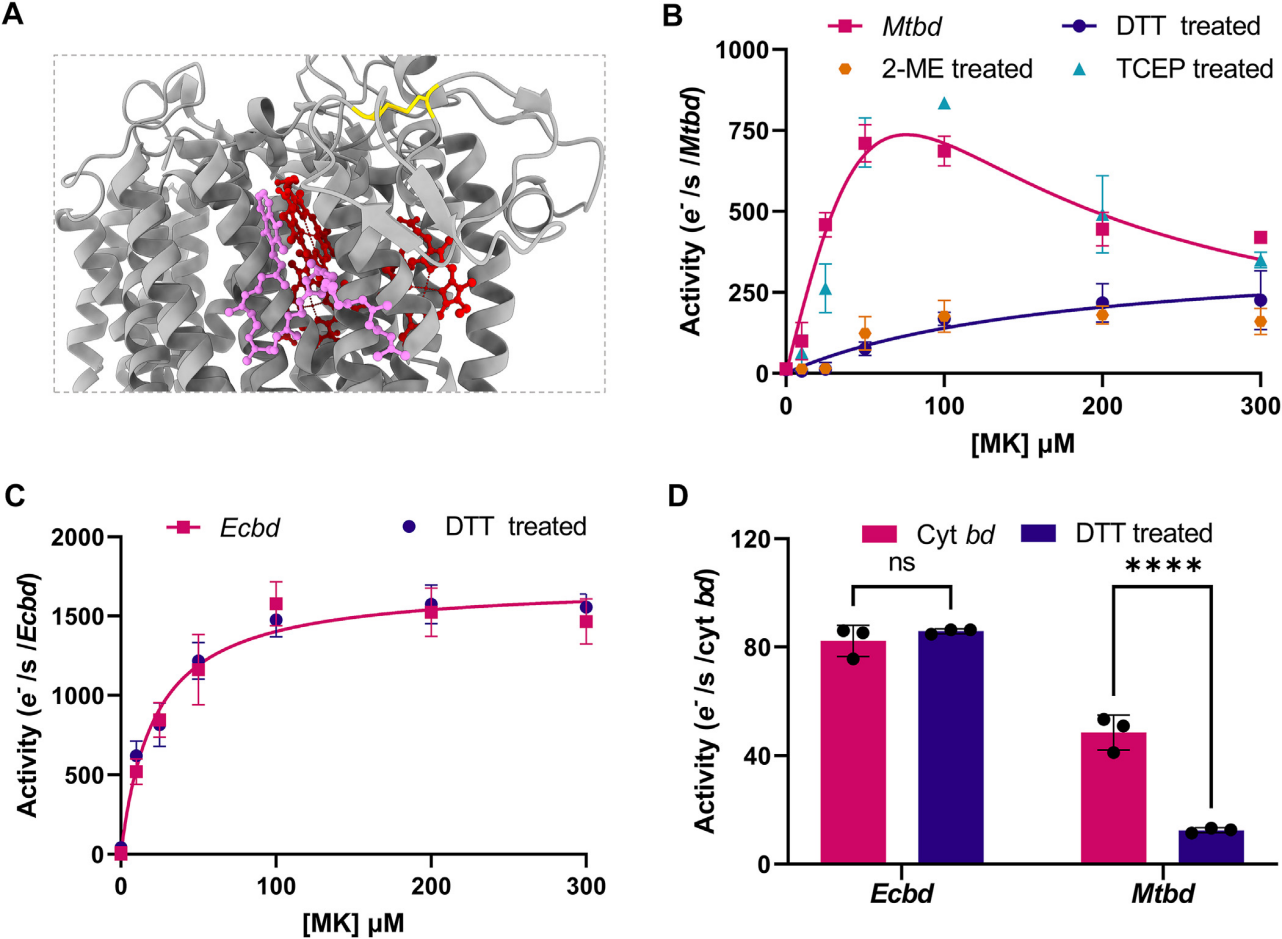
***Mtbd* regulation by the Q-loop disulfide bond.***A*, cryo-EM structure of *Mtbd* (Protein Data Bank code: 7NKZ) (14) with heme groups (*red*), MK-9 (*blue*), and unique Q-loop disulfide bond (*yellow*). The structure was analyzed using ChimeraX (70). *B*, *Mtbd* MK-1 kinetics in detergent micelles before and after treatment with TCEP (*blue*), DTT (*purple*), or 2-ME (*orange*). The reductants were maintained in the assay buffer to prevent the reformation of the disulfide bond. Significant inhibition of oxidase activity is observed after DTT or 2-ME treatment. *C*, *Ecbd* MK-1 kinetics in detergent micelles before and after DTT treatment. No difference is observed. *D*, oxidase activity of proteoliposomes containing *Mtbd* (0.25% MK-9) or *Ecbd* (1% MK-9) before and after DTT treatment. Significance is tested using an unpaired *t* test in GraphPad Prism. ∗∗*p* < 0.01. 2-ME, 2-mercaptoethanol; MK-9, menaquinone 9; *Mtbd*, cytochrome *bd* from *Mycobacterium tuberculosis*; TCEP, Tris(2-carboxyethyl)phosphine.

*Mtbd* is characterized by a unique disulfide bond that was hypothesized to reduce the flexibility of the substrate binding Q-loop (Fig. 5*A*) and thereby confer a regulatory role for enzyme activity (14). Previous studies examining this mechanism faced limitations, as they relied on chemical reductants, such as DTT, to reduce the quinone pool, which inadvertently affects the disulfide bond itself. Using NDH-2 to enzymatically reduce the quinone pool enables the comparison of *Mtbd* activity before and after incubation with chemical reductants to reduce the disulfide bond and probe its effects on *Mtbd* activity.

Incubation of *Mtbd* with either DTT or 2-mercaptoethanol (2-ME) showed a significant reduction in oxidase activity (Fig. 5*B*). In addition to the decrease in *Mtbd* activity, the disulfide bond breakage eliminates MK substrate inhibition, strongly suggesting that the reduction of the disulfide bond changes the structure or dynamics of the quinone-binding pocket. Unexpectedly, preincubation with the reductant Tris(2-carboxyethyl)phosphine (TCEP) did not decrease *Mtbd* activity, indicating that interactions with the disulfide bond might be hindered by surface accessibility or charge repulsion, as confirmed by nonreducing SDS-PAGE (Fig. S4). To verify that the chemical reductants only impact the disulfide bond and no other aspects of *Mtbd*, the same measurements were performed with *Ecbd*, which lacks the Q-loop disulfide bond (Fig. 5*C*). This confirms that there is no difference in *Ecbd* activity after treatment with DTT and consolidates the conclusion that the decrease in the activity of *Mtbd* stems from the reduction of the Q-loop disulfide bond.

To ensure that the observed effects are not artifacts of the detergent environment, the measurements were repeated in our proteoliposomal system and corrected for liposomal MK-9 auto-oxidation (Fig. S4*E*). Again, the *Ecbd* activity remains unchanged by the DTT treatment, whereas the *Mtbd* activity decreased by approximately 75% (Fig. 5*D*).

### Structural changes induced by the reduction of the Q-loop disulfide bond

**Figure 6:**
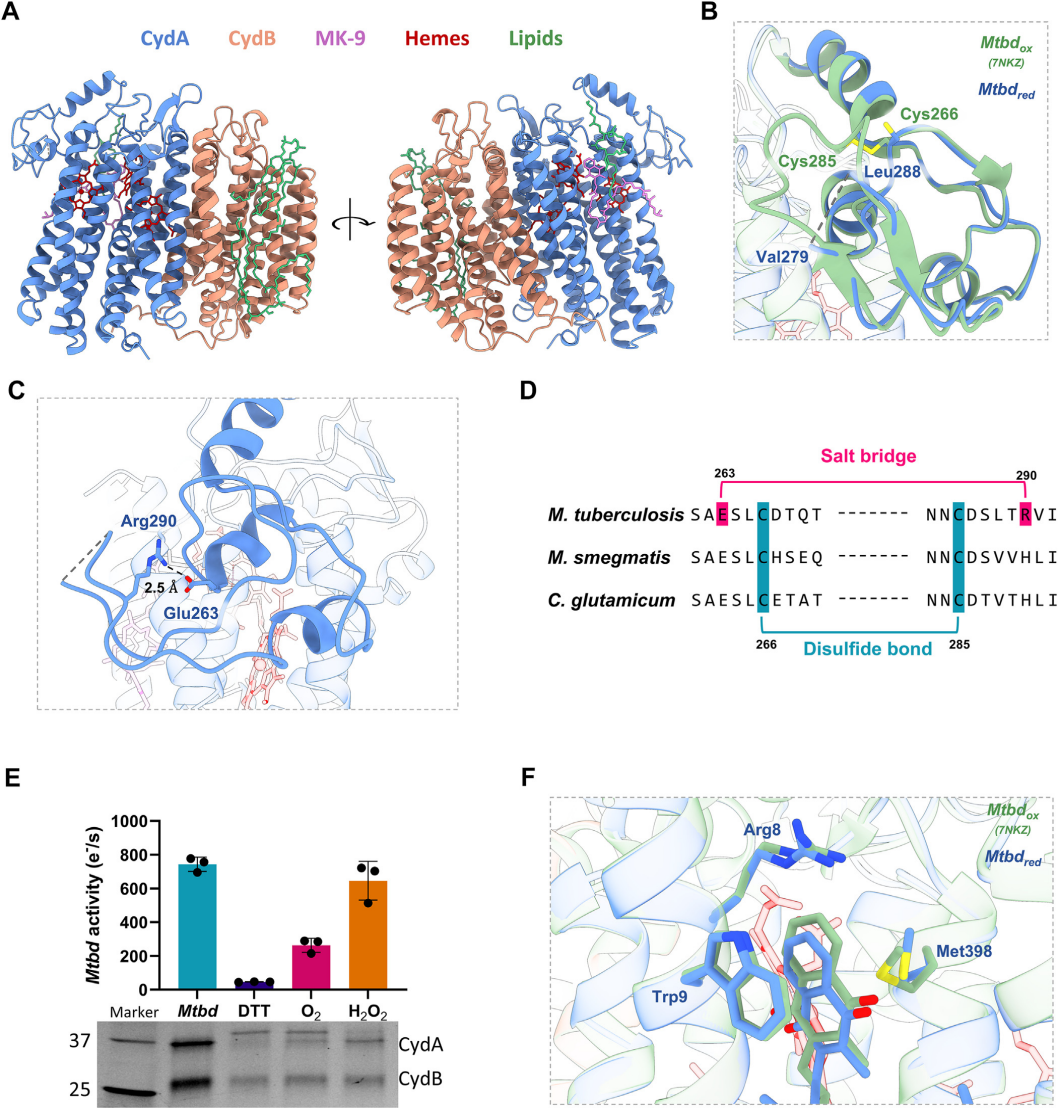
**Structural changes induced by reduction of the *Mtbd* Q-loop disulfide bond.***A*, cryo-EM structure of disulfide reduced *Mtbd*. *B*, increased local flexibility in the Q-loop after disulfide reduction, resulting in a density loss from V279 to L288. *C*, a salt bridge between Arg290 and Glu263 stabilizes the N-terminal part of the Q-loop. *D*, sequence alignment of the *Mycobacterium tuberculosis*, *Mycobacterium smegmatis*, and *Corynebacterium glutamicum* cyt *bd* Q-loop, showing the unique Arg290 participating in the stabilizing salt bridge. *E*, *Mtbd* kinetics after disulfide reduction. Exposure to ambient oxygen or 50 μM H_2_O_2_ for 30 min results in a regain of oxidase activity, together with a reformation of the bond. *F*, local changes in the MK9-binding pocket. Slight conformational changes are observed in Met398 and MK-9. MK-9, menaquinone 9; *Mtbd*, cytochrome *bd* from *Mycobacterium tuberculosis*.

To investigate the structural changes induced by the reducing conditions on *Mtbd*, single-particle cryo-EM analysis was performed. The reduced *Mtbd* structure was solved at a global resolution of 3.1 Å. This includes a 3 to 5 Å micelle, with the protein resolved at a resolution of 2.3 to 2.7 Å (Fig. S5). The reduced structure reveals notable structural changes in the Q-loop region and previously unstructured helix-connecting loops as well as a phosphatidylethanolamine (PE) lipid above the MK and two cardiolipin molecules (Fig. 6*A*).

The PE lipid is positioned on top of the MK-9-binding pocket, stabilized *via* hydrogen bonding with Arg8 (Fig. S6*A*). Interestingly, Arg8 has also been suggested as one of the stabilizing interactions binding to the MK headgroup (14). One cardiolipin phospholipid is bound to CydB, located in a similar position as the cardiolipin in cyt *bd*-II oxidase from *E. coli* (Protein Data Bank [PDB] code: 7OY2) (18). In addition, we assigned a second cardiolipin that is positioned superior to the first cardiolipin, occupying the same hydrophobic groove (Fig. S6*B*). Interestingly, mutations in this groove have been shown to affect *Mtbd* oxidase activity, further highlighting the stimulatory effect of cardiolipin (31, 37).

The overall RMSD of 0.39 Å between the oxidized (PDB code: 7NKZ (14)) and the disulfide-reduced structure indicates relatively minor global structural changes. However, increased local flexibility is observed for the *Mtbd* Q-loop residues 279 to 288, as evidenced by a weak electron density, suggesting a disordered conformation in the reduced state (Fig. 6*B*). To not bias our view of the Q-loop, we did not perform any micelle subtraction, as this could potentially remove electron density from the Q-loop region that is in close proximity to the micelle, leading to an inaccurate interpretation of the loop's structure and flexibility. Nevertheless, attempts to confidently model the Q-loop residues 279 to 288 were unsuccessful. The unstructured region within the Q-loop includes Cys285, which forms a disulfide bond with Cys266 under oxidized conditions. The especially weak electron density, as compared to the entire N-terminal region of the Q-loop, further confirms the breakage of this disulfide bond in the reduced structure.

In contrast to the homologous cyt *bd* oxidases in *Mycobacterium smegmatis* and *C. glutamicum*, where the entire N-terminal region of the Q-loop is disordered (15, 38), the *Mtbd* Q-loop remains partially structured. Although *bd* oxidases from *M. smegmatis* and *C. glutamicum* contain the conserved cysteine residues, they do possess a disulfide bond under the conditions tested (15, 38). This suggests that the *Mtbd* Q-loop possesses additional stabilizing interactions that help maintain a semistructured conformation even after disulfide reduction. This could be attributed to the presence of Arg290 in *Mtbd*, which forms a salt bridge with Glu263, thereby stabilizing the remaining Q-loop (Fig. 6*C*). Arg290 is unique to *Mtbd*, as the homologous cyt *bd* oxidases in *M. smegmatis* and *C. glutamicum* possess a histidine in the corresponding position (Fig. 6*D*) and could explain the fully unstructured N-terminal Q-loop observed in these homologs.

The salt bridge might help to maintain spatial proximity between Cys266 and Cys285 and facilitate the eventual reformation of the disulfide bond, suggesting a potential regulatory mechanism for *Mtbd* activity. Indeed, exposure of the reduced *Mtbd* to ambient oxygen concentrations resulted in a ∼30% recovery of activity, followed by near full recovery after exposure to 50 μM H_2_O_2_. The activity regain was accompanied by reformation of the disulfide bond, as confirmed by nonreducing SDS-PAGE (Fig. 6*E*).

Despite the local changes in the Q-loop upon disulfide reduction, MK-9 remains tightly bound near Trp9. A minor shift of Met397 is observed, increasing its distance to the MK-9 headgroup from 3.6 to 3.8 Å (Fig. 6*F*). In addition, we observe a slight shift of MK-9 out of the binding pocket, increasing its distance to Trp9 from 3.7 to 3.9 Å.

Importantly, however, the minor structural changes around MK-9 likely cannot account for the observed decrease in enzyme activity. We hypothesize that the observed MK-9 plays a structural role rather than serving as the substrate, which is supported by the cryo-EM density maps of *Mtbd* in the presence of the potent quinone-analog inhibitors AurD (14) and AD3-11 (39) (EMD-12532 and EMD-12533) (14). Despite a vast molar excess of these inhibitors, the MK-9 density remains unaltered, suggesting that the inhibitors occupy a different binding pocket while *Mtbd* is fully inhibited (14). This indicates the existence of a separate substrate binding site, potentially between heme *b*_558_ and the Q-loop, as hypothesized in other cyt *bd* oxidases (16, 17, 18).

## Discussion

Cyt *bd* is a critical part of the prokaryotic respiratory chain to maintain ATP regeneration under microaerobic conditions (3). *Mtbd* has been highlighted for its essentiality under oxygen-limiting conditions and its related interest as an antibiotic target against *M. tuberculosis* (5). Most current knowledge, however, comes from mechanistic studies on homologous enzymes such as *Ecbd* and *C. glutamicum* cyt *bd*, which have diverse structural features, such as a lack of the characteristic *Mtbd* Q-loop disulfide bond (14, 15, 16, 17).

The minimal respiratory chain, both in detergent and liposomes, unambiguously indicates that *Ecbd* can oxidize UQ, MK, and DMK. The affinity of *Ecbd* for UQ-1 (*K*_m_: 149 ± 26 μM) is in line with previously reported values (31, 35, 36, 40, 41), whereas the catalytic rate at 200 μM UQ-1 (1268 ± 81 *e*^−^ s^−1^) is higher than the literature-reported values (889 ± 30 *e*^−^ s^−1^) (16) and is attributed to the difference in the detergent environment and assay conditions. The fact that we see activity with MK is in direct contrast with an earlier study that suggests that *Ecbd* cannot oxidize MK (18). We note that in this earlier study, MK was reduced by DTT. Indeed, when we repeated the assay with DTT instead of NADH–NDH-2 to reduce MK, we did not observe any activity. Here, we propose that although DTT is a good reductant for UQ and hence a good reductant to measure oxygen-reducing activity by cyt *bd*, DTT might be a poor reductant and rate limiting when the assay is performed with MK. The observation that *Ecbd* can oxidize MK and DMK is consistent with the *in vivo* upregulation of MK and DMK during microaerobic respiration, conditions that also lead to increased expression of *Ecbd* (28, 29, 42).

The catalytic rates of both *Ecbd* and *Mtbd* were approximately 10-fold lower when using the natural lipophilic quinones in comparison to their often-used water-soluble analogs. Similar effects have been shown before, where small differences in quinone isoprenoid units altered turnover rate (43, 44). This highlights the need for physiologically relevant assay conditions to obtain appropriate kinetic rates and inhibition values. The difference in activity when using the native quinones could be explained by a difference in binding or diffusion kinetics, as many of the quinone-bound oxidase structures have shown that the quinone isoprenoid tails play a significant role in substrate–enzyme interactions (14, 45), and longer chains might result in slower substrate binding and release (46).

*Mtbd* exclusively catalyzes MK oxidation, while competition experiments suggest that UQ and DMK can bind to the same active site. A similar phenomenon was shown for cyt *bd* from *C. glutamicum*, and cytochrome *bcc* from *M. tuberculosis*, which also lacked oxidase activity using UQ as a substrate (34, 43). We postulate that this lack of UQ-1 turnover in *C. glutamicum* cyt *bd* is caused by a thermodynamic barrier between two-electron oxidation of UQ (100 mV) and the single-electron acceptor heme *b* (102 mV) (15, 47). Similar suggestions have been made for mycobacterial cytochrome *bcc*, where the heme redox potentials are too low to allow the oxidation of UQ (34, 48). Although the heme potentials for *Mtbd* are unknown, we postulate that the same thermodynamic barrier applies here.

*Mtbd* was unable to oxidize DMK, which is similar in structure to MK, and has a reduction potential that lies in between that of MK and UQ. In *M. tuberculosis*, DMK is a precursor in MK biosynthesis. Small-molecule inhibitors of MenG, the enzyme that converts DMK into MK, were bactericidal in *M. tuberculosis* (49, 50). This substantiates the inability of *Mtbd* to turn over DMK and highlights the tight specificity between the quinone pool and *Mtbd*. In addition, this emphasizes that inhibition of MK biosynthesis is a valid antibiotic strategy that targets the fundamental electron transfer steps between the respiratory chain components.

In contrast to our observation reported here, *Mtbd* expressed in *E. coli* has been reported to oxidize UQ when assayed in crude membrane extracts (44). Potentially, the observed UQ oxidation was caused by interfering oxidases present in the membrane extracts, such as *E. coli* cyt *bd*-II. Further studies would be required to determine why the assay in crude *E. coli* membrane extracts gives rise to different results than *Mtbd* purified from *M. smegmatis*, as reported here.

Based on our structural analysis of *Mtbd*, we suggest that the observed MK-9 in the binding pocket adjacent to Trp9 confers a structural role rather than acting as the substrate under the conditions tested. The published structure (14) and our structure of the disulfide reduce *Mtbd*, do not indicate an alternative quinone substrate-binding site, and it is therefore critical to uncover and prove the cyt *bd* quinone substrate-binding pocket in the future. While the location of the *Mtbd* catalytic site remains speculative, studies on the homologs *Ecbd* (16) and *Ecbd*-II (5) have indicated binding at the N-terminal part of the Q-loop, stabilized by Arg245 near heme b_585_. Whether these results translate to *Mtbd* remains unclear. The binding of a quinone substrate might be correlated to structural changes in which the disulfide bond has a more pronounced role, possibly explaining the role of the disulfide bond on the activity of *Mtbd*.

The *Mtbd* disulfide bond is unique among the currently studied cyt *bd* oxidases. Despite the conservation of these cysteine residues in *M. smegmatis* (38) and *C. glutamicum* (15) cyt *bd*, these proteins do not show a formed cysteine bond in their structures under the conditions tested. Here, we show that the chemical reduction of the *Mtbd* disulfide bond results in a significant decrease in oxidase activity. Molecular dynamics studies previously suggested that reduction of the *Mtbd* disulfide bond greatly increases Q-loop flexibility (14). In contrast, the structure of the disulfide-reduced *Mtbd* indicates only minor structural changes outside the Q-loop, whereas the Q-loop itself also remains mostly structured except for residues 279 to 288. In the structure of the disulfide-reduced state, the cysteines remain in relatively close proximity, possibly aided by a salt bridge between Arg290 and Glu263, which could explain why the disulfide bond in *Mtbd* can fully (re)form upon exposure to hydrogen peroxide.

This redox sensory role of the Q-loop disulfide bond might contribute to a rapid adaptation of *M. tuberculosis* when exposed to the hostile conditions encountered during infection. We hypothesize that *Mtbd* is expressed and maintained in the disulfide-reduced state, allowing for efficient respiration using the cytochrome *bcc*:*aa*_3_ branch. This is in line with our results, showing that *Mtbd* maintains mostly reduced upon exposure to ambient oxygen. This primes *Mtbd* to be activated upon exposure to the ROS encountered during phagocytosis. Our hypothesis could also explain the instantaneous increase in respiration by *M. tuberculosis* upon inhibition of the *bcc:aa*_*3*_ supercomplex (20), which is shown to also increase periplasmic ROS. The disulfide bond of *Mtbd* might be further regulated by the large network of proteins and small molecules controlling *M. tuberculosis* redox homeostasis (51, 52). How this regulatory mechanism would play into the larger metabolic response of *M. tuberculosis* to ROS remains unknown, especially in the rapid time frames *Mtbd* shows to be activated (21).

To our knowledge, this is the first proof of a terminal oxidase under the regulation of a redox-sensing disulfide bond. Enzyme-level regulation of *Mtbd* with different redox pressures might be important for *M. tuberculosis* to reroute electrons from cytochrome *bcc*:*aa*_3_ to *Mtbd*, rapidly adapting to environmental changes. Potentially, this aids in the survival in ROS-rich environments during infection (53, 54), with the suggested role of *Mtbd* in hydrogen peroxide resistance (55). In addition, *Ecbd* has been shown to actively detoxify the cellular environment from hydrogen peroxide (56) and peroxynitrite (57), indicating potential additional roles for *Mtbd* as a survival factor for *M. tuberculosis*. Future research should focus on the physiological role and regulation of the *Mtbd* disulfide bond and its influence on *M. tuberculosis* survival. Additional investigations of the interplay between *Mtbd* and environmental redox pressures, especially regarding its potential detoxifying role as seen with *Ecbd* (56), will offer valuable insights into the adaptive role of *Mtbd* in the defense against ROS and antibiotic compounds encountered during infection.

## Experimental procedures

### Expression and purification of *E. coli* cyt *bd*-I

The expression of *E. coli* cytochrome *bd*-I (*Ecbd*) was performed as previously described (58), with slight modifications. Briefly, MB43 cells (59, 60) transformed with pET17b-CydABX-linkerstreptag were grown overnight in LB with 100 μg/ml ampicillin (250 RPM, 37 °C). The culture was diluted to an absorbance of ∼0.1 in LB ampicillin and grown to an absorbance of ∼0.4. Expression was induced by the addition of 0.45 mM IPTG and carried out until an absorbance of ∼2.0. Cells were harvested by centrifugation (6371 rcf, 20 min, 4 °C) and resuspended in 50 mM Mops, pH 7.4, 100 mM NaCl, cOmplete EDTA-free Protease Inhibitor (ROCHE), at 1 g wet cells per 5 ml buffer. Cells were disrupted by a single pass through a Stansted pressure cell homogenizer (270 MPa). Unbroken cells were pelleted and discarded by centrifugation (10,000 rcf, 20 min, 4 °C). Crude membranes were isolated by ultracentrifugation (200,000 rcf, 1h, 4 °C) and resuspended to 10 mg/ml protein concentration in 50 mM Mops, 100 mM NaCl, pH 7.4. Detergent extraction of the membrane proteins was performed by incubation with 0.5% lauryl maltose neopentyl glycol (LMNG) for 1 h at 4 °C with gentle mixing. Insoluble material was pelleted and discarded by ultracentrifugation (200,000 rcf, 30 min, 4 °C) followed by application of the soluble fraction to a StrepTrap HP column (Cytiva) at 1 ml/min. To remove unbound proteins, the column was washed with 50 mM sodium phosphate, 300 mM NaCl, 0.005% LMNG, pH 8.0. Elution was performed by the addition of 50 mM sodium phosphate, 300 mM NaCl, 2.5 mM desthiobiotin, 0.005% LMNG, pH 8.0, after which purity was confirmed by SDS-PAGE. Fractions containing pure *E. coli* cyt *bd*-I were pooled, concentrated, and stored at −80 °C until further use.

### Expression and purification of *M. tuberculosis* cyt *bd*

*Mtbd* expression and purification were performed as previously described with slight modifications (14). *M. smegmatis* MC (2) 155 ΔCydAB (61) was transformed with the pLHCyd plasmid using electroporation with an Eppendorf eporator (2.5 kV). Positive transformants were selected by plating the cells on LB agar with 50 μg/ml hygromycin. A starter culture was inoculated in LB–hygromycin and grown for 72 h (250 RPM, 37 °C). The culture was diluted 1:100 and grown for an additional 72 h (200 RPM, 37 °C) until the cells were harvested (6371 rcf, 20 min, 4 °C) and resuspended in a fivefold volume of 50 mM Tris–HCl (pH 7.4), 5 mM MgCl_2_, 0.05% Tween-80, and cOmplete EDTA-free Protease Inhibitor. The cells were lysed by a double pass through a Stansted pressure cell homogenizer (270 MPa). Cell debris was pelleted and discarded by centrifugation at 10,000 rcf, 20 min at 4 °C. The crude membranes were extracted by ultracentrifugation (200,000 rcf, 1 h, 4 °C) and resuspended in 20 mM Tris–HCl, pH 7.4, 0.05% Tween-80, and 10% glycerol to a total protein concentration of 10 mg/ml. The proteins were solubilized by the addition of 0.5% LMNG with gentle mixing (1 h, 4 °C). Insoluble material was pelleted by ultracentrifugation (200,000 rcf, 30 min, 4 °C) and discarded. The soluble fraction was incubated overnight at 4 °C with Pierce Anti-DYKDDDDK Affinity Resin (ThermoFisher) and purified according to the manufacturer’s instructions. Briefly, the flow through was removed by centrifugation at 1000 rcf, followed by four washing steps with 10 colume volumes of 50 mM Tris–HCl (pH 7.4), 150 mM NaCl, and 0.005% LMNG. The protein was eluted by the addition of 50 mM Tris–HCl (pH 7.4), 150 mM NaCl, 0.02% dodecyl beta-d-maltoside (DDM), 1 mg/ml 3X-FLAG peptide (Genscript). Final purification was achieved by SEC on a Superdex 200 increase 10/200 column at 0.5 ml/min (50 mM Tris–HCl [pH 7.4], 150 mM NaCl, and 0.005% LMNG). SEC peak fractions were evaluated on SDS-gel, and pure *Mtbd* was pooled, concentrated, and stored at −80 °C until further use.

### Expression and purification of NDH-2

The gene for *C. thermarum* NDH-2 with an N-terminal hexahistidine tag was ordered from GeneArt and cloned in the pET28 vector between NcoI and XhoI, giving rise to the construct pET28-NDH-2_NtermHis. pET28-NDH-2_NtermHis was transformed into C41 (DE3) cells and plated on LB kanamycin to select positive transformants.

Expression and purification were performed based on the procedure from Heikal *et al.* (62) with slight modifications. Briefly, a streak of transformants was inoculated and grown overnight (250 RPM, 37 °C). The overnight culture was diluted to absorbance of 0.1 in LB kanamycin and grown to an absorbance of 0.5 before induction with 0.25 mM IPTG. Expression was carried out for 4 h before cells were harvested (6371 rcf, 20 min, 4 °C). The cells were resuspended in a fivefold volume of 50 mM Tris–HCl, 5 mM MgCl_2_, pH 8.0, and lysed by a single pass through a Stansted pressure cell homogenizer (270 MPa). Unbroken cells were pelleted by centrifugation (10,000 rcf, 20 min, 4 °C) and discarded. Crude membrane fractions were pelleted by ultracentrifugation (200,000 rcf, 1 h, 4 °C) and resuspended at a 10 mg/ml total protein concentration in Tris–HCl, 150 mM NaCl, and 20 mM imidazole. Membrane proteins were extracted by treatment with 1% DDM for 1 h at 4 °C with gentle mixing. The membranes were removed by ultracentrifugation (200,000 rcf, 30 min, 4 °C) followed by application of the soluble fraction to a HiTrap Nickel NTA column (Cytiva). The unbound proteins were washed from the column with washing buffer (50 mM Tris–HCl [pH 8.0], 150 mM NaCl, 20 mM imidazole, and 0.02% DDM) followed by elution using stepwise addition of elution buffer (50 mM Tris–HCl, 150 mM NaCl, 500 mM imidazole, and 0.02% DDM). NDH-2 eluted at approximately 30% elution buffer, as confirmed by SDS-PAGE gel and Western blot. Final purification was achieved by SEC on a Superdex increase 200 10/300 column (Cytiva) at 0.5 ml/min (50 mM Tris–HCl [pH 8.0], 500 mM NaCl, 5% glycerol, and 0.02% DDM). Pure fractions were pooled, concentrated, and stored at −80 °C until further use.

### Cyt *bd* reconstitution in proteoliposomes

Lipids were purchased from Avanti Polar Lipids and used as received. A lipid mixture of POPE:POPG:CA (60:30:10 for *Ecbd* proteoliposomes, 30:60:10 for *Mtbd* proteoliposomes), enriched with the desired concentrations of UQ-10 (Sigma) or MK-9 (Cayman Chemical), was dried under a stream of nitrogen. Final traces of CHCl_3_ were removed overnight under vacuum. The lipid film was rehydrated and resuspended to a final concentration of 10 mg/ml in 20 mM Mops, 30 mM Na_2_SO_4_, 100 mM KCl, pH 7.4, by vortexing. Cyt *bd* reconstitution was performed as described (63). Briefly, cyt *bd* was added to the liposome solution at 1 w/w% protein/lipids and mixed for 30 min by inversion at room temperature. Insoluble materials were removed by centrifugation in an Eppendorf tabletop centrifuge (14,100 rcf, 5 min). The proteoliposome cyt *bd* concentration was determined by redissolving a sample in 2% octyl-β-glucoside, followed by quantification of the Soret band with the corresponding extinction coefficient (*Ecbd*: ε_417_ 230 mM^−1^ cm^−1^ (64), *Mtbd*: ε_414_ 279 mM^−1^ cm^−1^). The latter extinction coefficient of the *Mtbd* Soret band (414 nm) was determined from UV–vis absorbance in relation to protein concentrations determined by bicinchoninic assay from three different protein preparations. The extinction coefficient (ε_414nm_ = 279 ± 13 mM^−1^ cm^−1^) was comparable to other *bd*-type oxidases (29).

### Enzyme kinetics with water-soluble quinone analogs

Oxygen consumption of LMNG-solubilized cyt *bd* with quinone analogs was measured on an oxygraph (Hansatech Ltd) system at 20 °C. The quinone analogs, UQ-1 (Sigma), deoxylapachol (DMK-1; MedChem Express), or MK-1 (Santa Cruz Biotech), were added to the reaction chamber at the desired concentration in 50 mM Mops (pH 7.0), 150 mM NaCl, and 0.005% LMNG. Quinone-mediated auto-oxidation was determined by enzymatic reduction of the quinones by *C. thermarum* NDH-2 (30 nM) after the addition of 1 mM NADH. Oxygen consumption was initiated by the addition of cyt *bd* (4 nM for *Ecbd* and 6.5 nM for *Mtbd*). The enzyme activity was measured by subtraction of the quinone auto-oxidation rate from the initial slope after cyt *bd* addition. The kinetics curves were fit, and where possible, enzymatic parameters were determined using GraphPad Prism (GraphPad Software, Inc) using either Michaelis–Menten equation (Equation 1) or substrate inhibition kinetics (Equation 2).

(1) $$V_{i}=\frac{K_{cat}\left[E\right]\left[S\right]}{K_{m}+\left[S\right]}$$

(2) $$V_{i}=\frac{K_{cat}\left[E\right]\left[S\right]}{K_{m}+\left[S\right]\frac{1+\left[S\right]}{K_{i}}}$$

### Cyt *bd* oxygen consumption in proteoliposomes

Oxygen consumption was measured on an oxygraph system at 20 °C. Prior to the measurement, cyt *bd* proteoliposomes were diluted to the desired concentration (20 nM cyt *bd*) in 50 mM Mops, 150 mM NaCl, pH 7.0. The proteoliposomes were incubated for 30 min at room temperature with 100 nM NDH-2 to complete the proteoliposomal system. Oxygen consumption was initiated by the addition of 1 mM NADH. The oxygen consumption rate was quantified using the initial slope after NADH addition and corrected for auto-oxidation from cyt *bd*-free liposomes.

### Cyt *bd* oxygen consumption after treatment with reductants

The effect of *Mtbd* Q-loop disulfide bond reduction was studied by 30 min of preincubation with chemical reductants (10 mM TCEP, 10 mM DTT, or 100 mM 2-ME). When required, the reductant was removed by spin filtration before exposure to either ambient oxygen concentration or 50 μM H_2_O_2_ for 30 min. The samples were diluted to the concentrations used previously in the LMNG-solubilized or proteoliposomal measurements. The buffer was supplemented with the respective chemical reductant to maintain the reductive environment during the measurement (1 mM TCEP [10 mM showed auto-oxidation]), 10 mM DTT, and 100 mM 2-ME). The measurements were performed as mentioned previously.

### Nonreducing SDS-PAGE

The effects of environmental redox potentials on the *Mtbd* disulfide bond were determined by nonreducing SDS page, omitting reductant from the loading buffer. *Mtbd* was exposed to the desired amount of reductant (10 mM DTT) for 1 h, before removal by spin filtration. If required, *Mtbd* was exposed to ambient oxygen concentrations or 50 μM H_2_O_2_ for 30 min before denaturation in SDS-PAGE buffer without additional reductant. Shifts in the *Mtbd* CydA protein band were determined by nonreducing SDS-PAGE gel.

### Cryo-EM sample preparation and data collection

C-flat R1.2/1.3-Cu 300 mesh grids (Electron Microscopy Sciences) were freshly glow discharged with PELCO easiGlow device at 15 mA for 90 s. LMNG-solubilized *Mtbd* was pretreated with 4 mM DTT for 1 h to break the Q-loop disulfide bond, followed by the application of 4 μl sample (2.5 mg/ml) on the grid. Sample blotting was performed for 3 s, at 20 blot force using a Vitrobot IV device (ThermoFisher Scientific) operating at 4 °C and 100% relative humidity, directly before plunge freezing into liquid ethane.

A total of 14,078 movies were collected on a Titan Krios G1 (ThermoFisher Scientific) operating at 300 kV, equipped with a Gatan K3 detector and BioQuantum energy filter with a slit width of 20 eV. Movies were acquired in electron counting mode using aberration-free image-shift in EPU (ThermoFisher Scientific). A total dose of 100 e/Å2 with 100 frames, at 105,000× magnification with a calibrated pixel size of 0.836 Å and a defocus range of 1.0 to 2.0 μm.

### Cryo-EM data analysis

CryoSPARC live (65) was used for on-the-fly processing of data with motion correction using patch motion correction, contrast transfer function (CTF) estimation using patch CTF estimation, and particle picking based on Blob-picker using minimum/maximum diameters and filtering based on normalized cross-correlation score and Power score. Curated micrographs were imported into the regular version of CryoSPARC (version 4.3.1) for further processing. After importing filtered micrographs and 2D class templates from the live session, Template-picker was used to fine-tune the picking parameters. Multiple rounds of 2D classification were run to clean junk particles. An unsupervised *ab initio* map was built, and the map and extracted particles were taken further to multiple rounds of heterogeneous refinement. Only the best particles along with all volumes were taken to further heterogeneous refinement jobs until particles became constant in the best class. Particles from the best class were taken to nonuniform refinement jobs for global and local CTF refinements along with other high-order aberration corrections (66). Movies were taken to another motion correction job to remove the last 50 frames, in order to check for any particle damage from relatively higher electron dose in the later frames. These movies and particles from last refinement job were used to re-extract with the original box size. This was further taken to several more rounds of nonuniform refinements. Data processing was finished by achieving a high-resolution subatomic map of 2.96 Å. During the data processing, LMNG micelle was not subtracted from the maps, so as not to create any signal biasedness between detergent micelle and flexible parts of the protein.

### Model building

Model building was done using *Mtbd* model from 7NKZ and fitting main chains using ARP/wARP server. After the main chain fitting, all side chains were manually checked and fitted, and missing loops and termini were added in Coot software (version 0.9) (67), and missing residues from loops, N and C termini were added. Cardiolipins and PE were added to the designated density in *Mtbd*. Models and the corresponding maps were assessed and analyzed in ChimeraX (version 1.7.1) (68).

## Data availability

The cryo-EM map file of disulfide-reduced *Mtbd* was deposited in the EMD database and can be found under accession number EMD-50520. The model file of *Mtbd* was deposited to the PDB under 9FKA.

## Supporting information

This article contains supporting information (23, 24, 69).

## Conflict of interest

The authors declare that they have no conflicts of interest with the contents of this article.
