# Supplementary material for: Menaquinone-specific turnover by Mycobacterium tuberculosis cytochrome bd is redox regulated by the Q-loop disulfide bond
Source: J Biol Chem. 2024 Dec 18;301(2):108094. doi: 10.1016/j.jbc.2024.108094 (PMC11786768; doi:10.1016/j.jbc.2024.108094)
Supplement: Supporting information [file mmc1.docx]

Supplementary information

**Menaquinone-specific turnover by *M. tuberculosis* cytochrome *bd* is redox regulated by the Q-loop disulfide bond**

Tijn T. van der Velden^1^, Kanwal Kayastha^1^, Caspar Y. J. Waterham^1^, Steffen Brünle1, Lars J. C. Jeuken^1,*^

*^1^ Leiden Institute of Chemistry, Leiden University, PO Box 9502, 2300 RA, Leiden, The Netherlands*

** Corresponding author: L.J.C.Jeuken@lic.leidenuniv.nl*


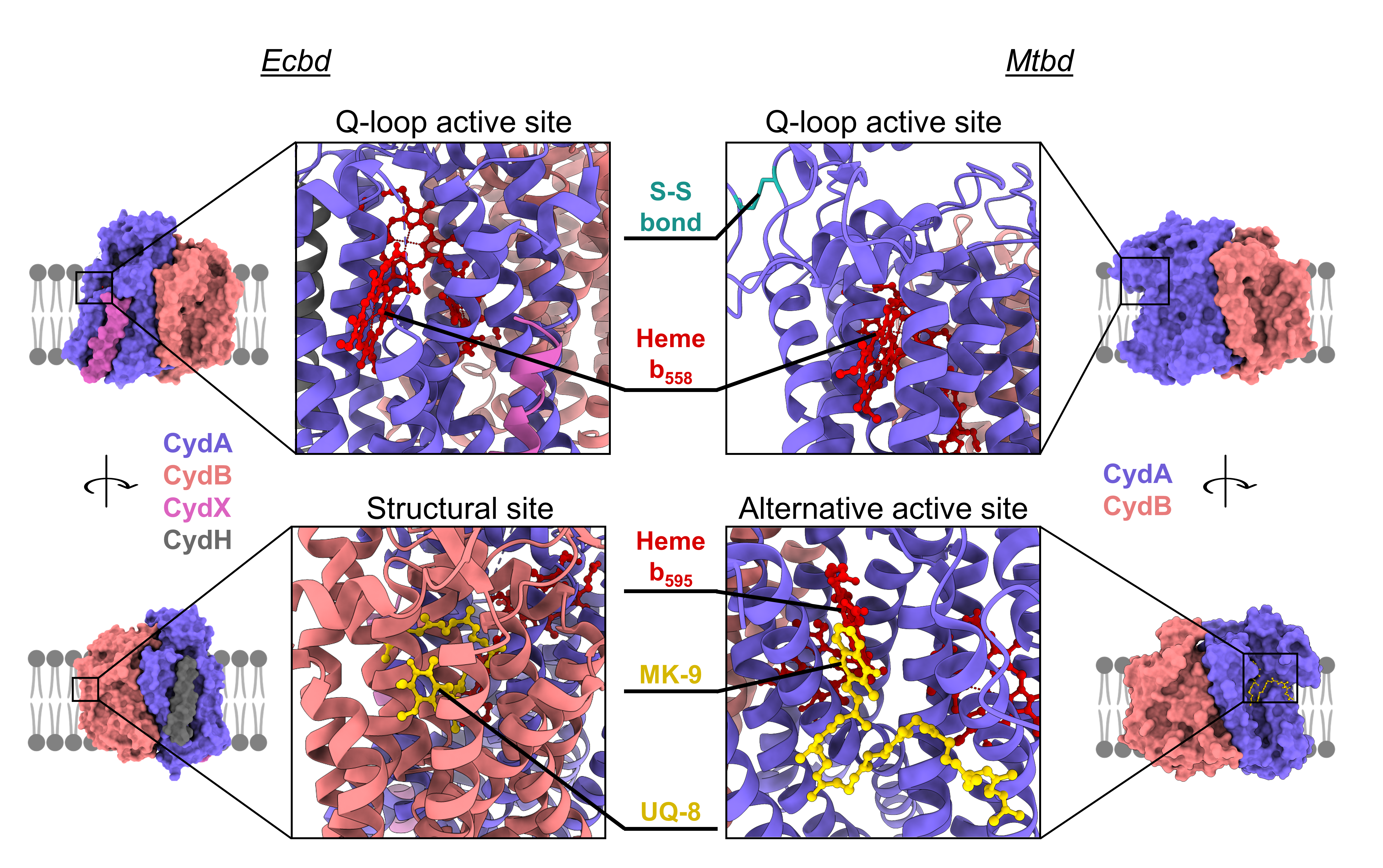


**SI Figure 1: Cryo EM strucures of Ecbd (PDB: 6RKO) and Mtbd (PDB: 7NKZ) with active and structural sites indicated.**


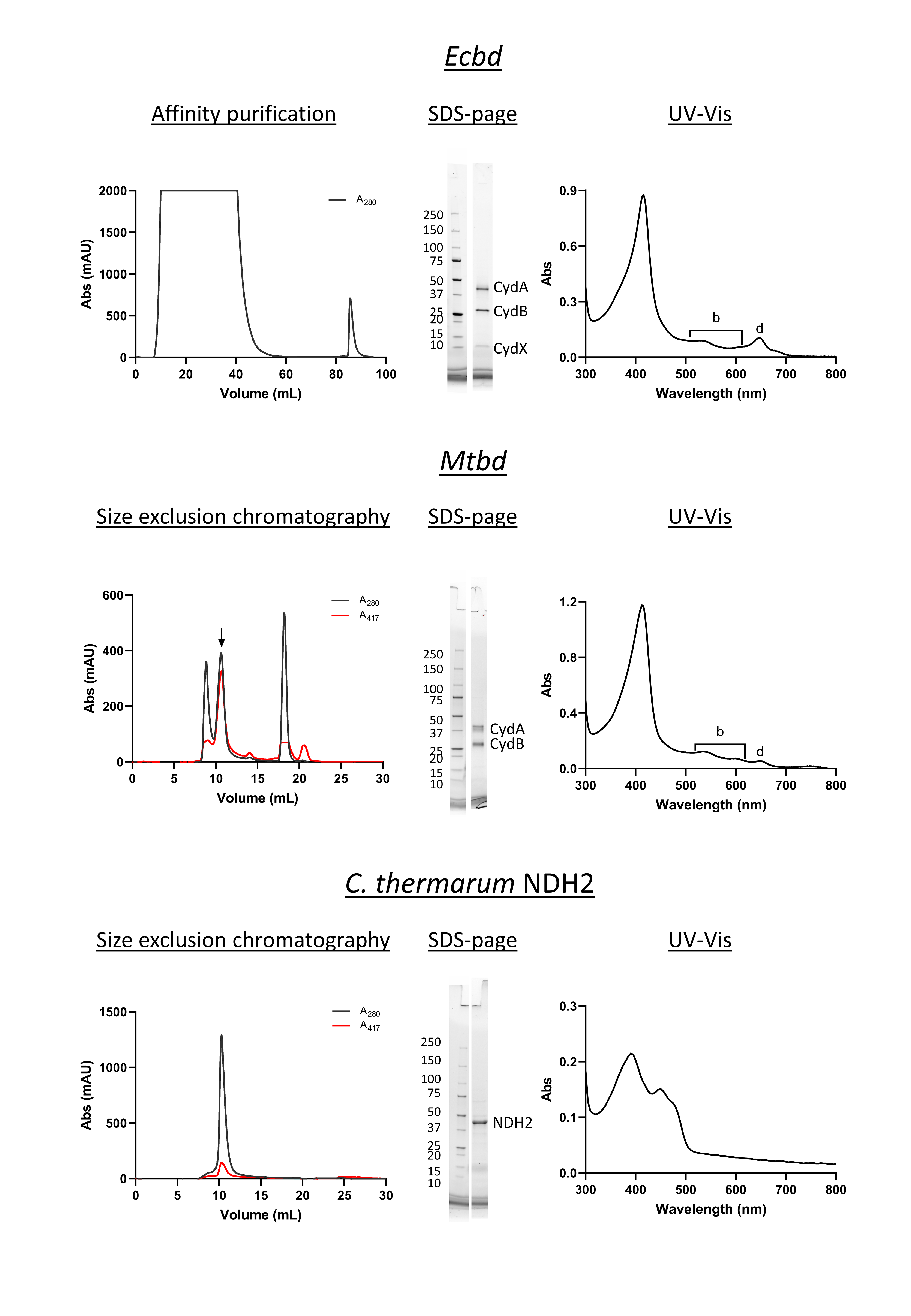


**SI Figure 2:** Purification, SDS-Page (TGX stain), and UV-Vis spectra of the isolated Ecbd, Mtbd and C. thermarum NDH2.


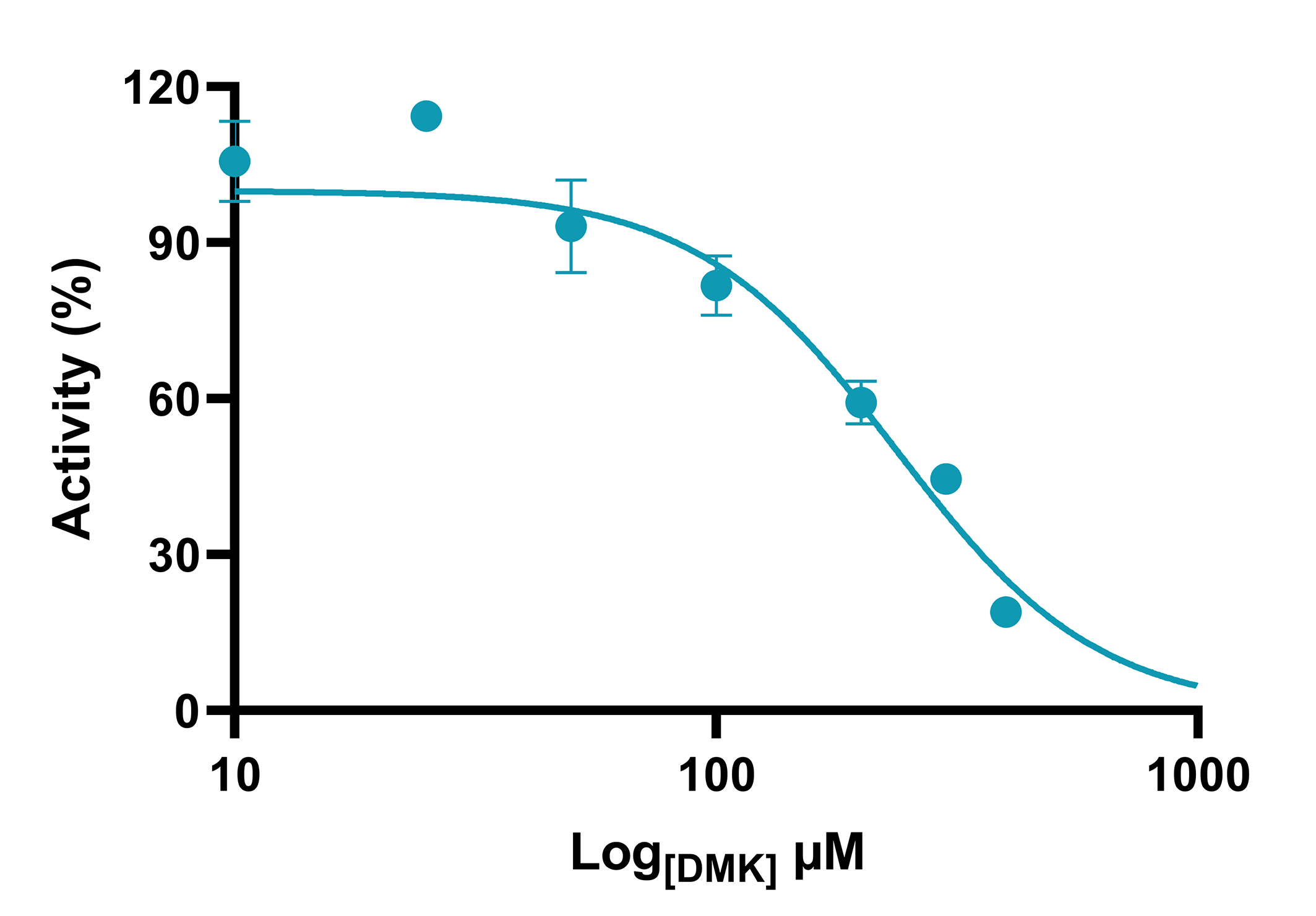


**SI Figure 3:** Inhibition of Mtbd (50 µM MK-1) by increasing DMK-1 concentration.


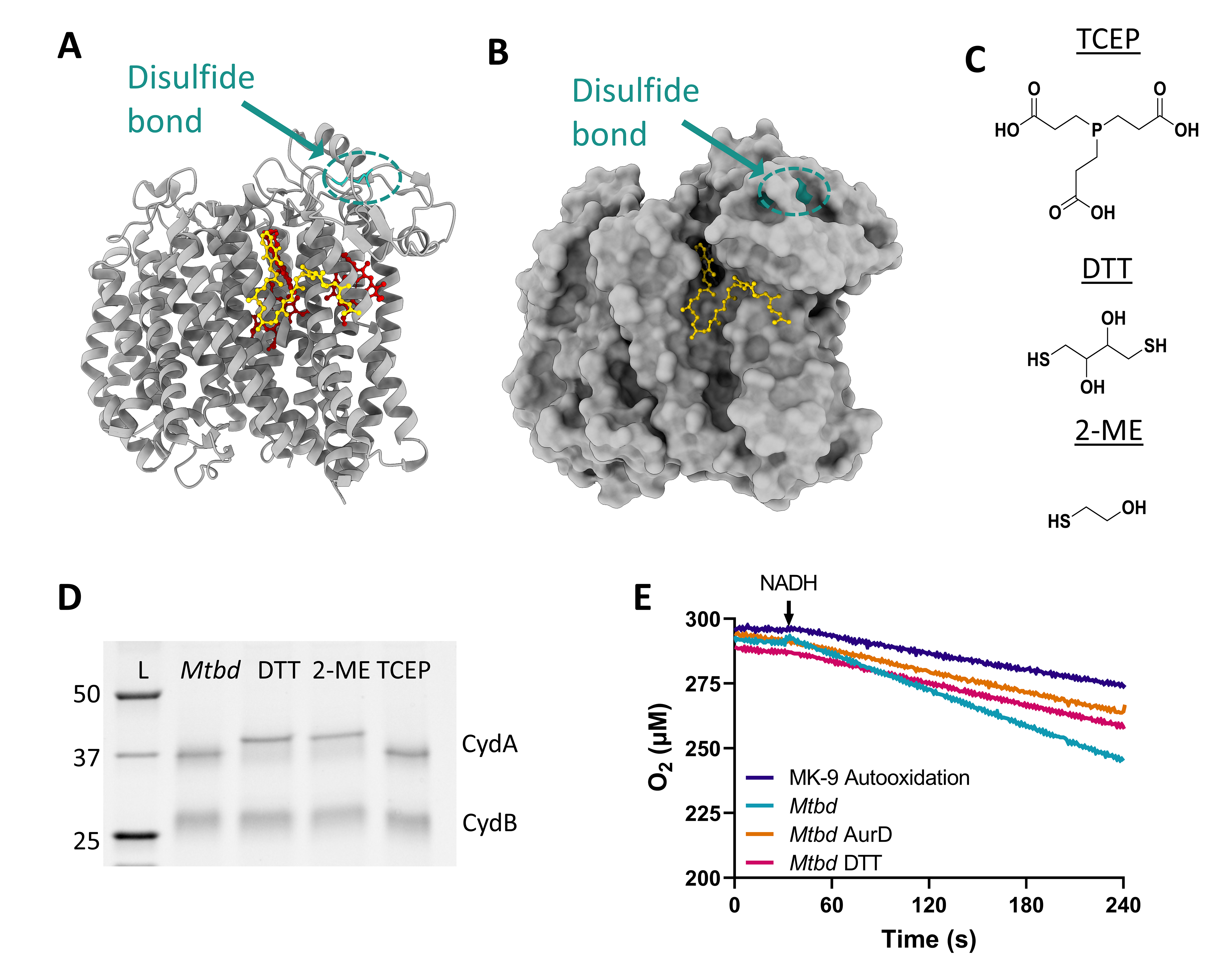


**SI Figure 4:** Accessibility of the Mtbd disulfide bond. Cartoon (**A**) and surface (**B**) representation of Mtbd with hemes (Red), MK-9 (yellow) and disulfide bond (blue). (**C**) chemical structure of the reductants used in this study. (**D**) Non-reducing SDS page (TGX stain) of Mtbd with prior treatment with different reductants. (**E**) Representative oxygraph traces of Mtbd liposomes (10 nM) before and after DTT treatment and inhibited by AurD, and MK-9 autoxidation trace of Cytbd free liposomes.

**Calculation of *E. coli* membrane quinone content**

| **Variables** | **Value** | **Ref** |
| --- | --- | --- |
| UQ content *E. coli* | 1.5 x 10^3^ nmol/g dry cell weight | ^1^ |
|  | 3.6 x 10^2^ nmol/g dry cell weight | ^2^ |
| *E. coli* dry weight | 3.0 x 10^-13^ gram per cell | ^3^ |
| Number of lipids per *E. coli* cell | 2.2 x 10^7^ lipids | ^3^ |
| Avogadro’s number | 6.022 x 10^23^ |  |

Calculation

Number of lipid per cell: 2.2 x 10^7^ / 6.02 x 10^23^ = 3.6 x 10^-17^ mol/cell

Number of lipids in 1 gram cells: 3.6 x 10^-17^ mol/cell × (1/3.0 x 10^-13^ g/cell) = 1.2 x 10^5^ nmol/g

% UQ in membrane^1^: = 1.5 x 10^3^ nmol/g / 1.2 x 10^5^ nmol/g × 100% = 1.23%

% UQ in membrane^2^: = 3.6 x 10^2^ nmol/g / 1.2 x 10^5^ nmol/g × 100% = 0.30%

Assuming a surface area of POPC to be 65 Å^2^ (MW = 760 g/mol) and a lipid bilayer thickness of 4 nm, 1% (w/w) UQ-10 (MW = 865 g/mol) is equivalent to 11 mM

Volume of 1 mol POPC bilayer:

(6.022 x 10^23^ × 0.65 nm^2^ x 4 nm) / 2 = 7.8 × 10^23^ nm^3^ = 0.8 L

1% (w/w) UQ-10 of 1 mol POPC is = 0.01 × 760 g = 7.6 g or (7.6 g / 865 g/mol) = 8.8 mmol

8.8 mmol / 0.8 L = 11 mM


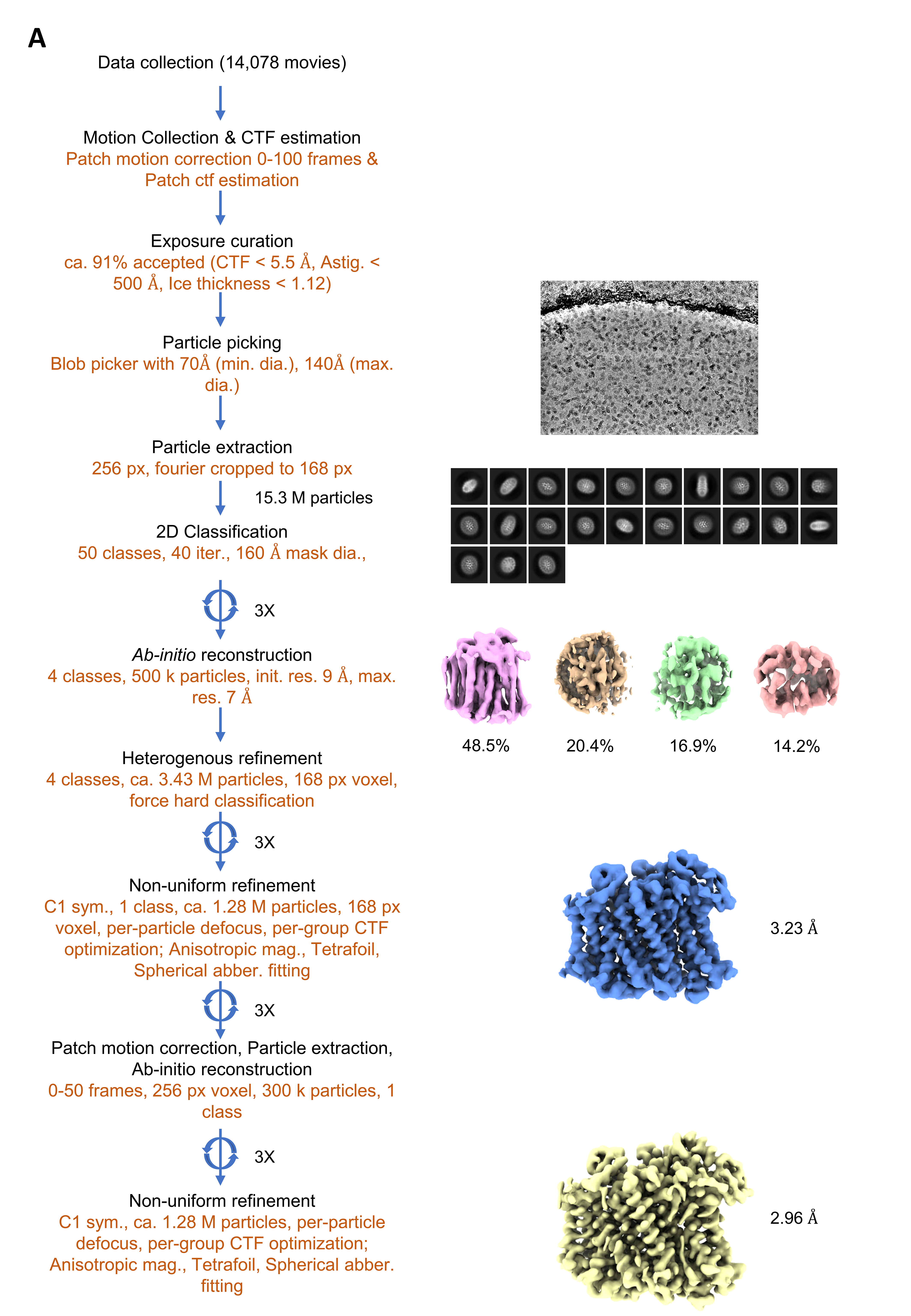


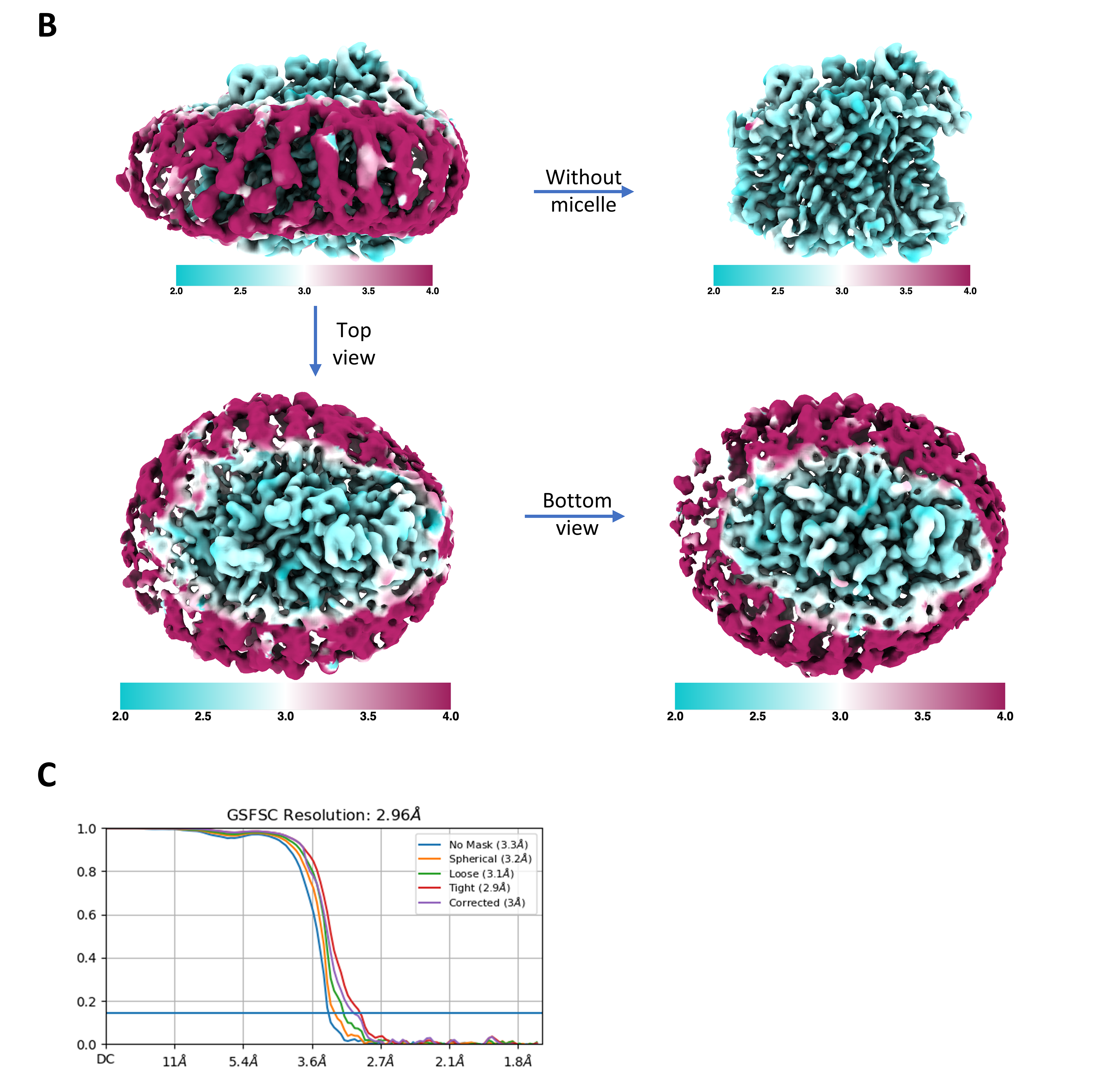


**SI Figure 5: CryoSPARC cryoEM data processing scheme.** (**A)** on the left side starting from importing movies, motion correction, ctf estimation for pre-processing steps along with curating exposures based on ctf resolution, astigmatism, and relative ice thickness. This was followed by particle-picking using blob picking based on particle diameter, inspecting picked particles, extracting and Fourier-cropping particle box to a smaller size of 168 pixels, to save processing time. Approximately 15.3 million picked particles were subjected to 3 rounds of 2D classification, followed by ab-initio reconstruction and taking all volumes and selected particles for 3 rounds of heterogeneous refinement to refine and sort particles into best 3D classes simultaneously. The best particle class and volume were further taken for 3 rounds of non-uniform refinements. A total of ca. 1.28 million particles yielded in a 3.32 Å reconstruction. From 100 total frames used in all processing until this step, the last 50 frames were cut off to process only the first 50 frames to check map quality and resolution improvements. Also, particles were re-extracted to the full box size of 256 pixels to check for any further resolution improvements. 3 more rounds of non-uniform refinements to optimize per-group ctf estimation, per-particle defocus, and fit spherical aberration, tetrafoil, anisotropic magnification, were performed before finalizing the cryo-EM map of 2.96 Å. On the right side of the scheme, there is an exemplary micrograph to show particle distribution. This is followed by selected 2D classes and then 4 ab-initio classes with increasing to decreasing order or particle distribution. Only the single highest class with 48.5 % particles was taken for further processing after careful inspection of all classes in ChimeraX. (**B)** Local resolution estimations between the resolution of 2 Å (teal) to 4 Å (Bordeaux). The first 3 figures counterclockwise show estimates along with LMNG detergent micelle from the side view (top left), top view (bottom left), bottom view (bottom right), and the fourth figure on the top right shows estimates at a threshold of 0.0637 to hide LMNG micelle and show local resolution for just protein parts. (**C)** FSC curve of the final reconstruction at a resolution of 2.96 Å from gold-standard FSC of 0.143.


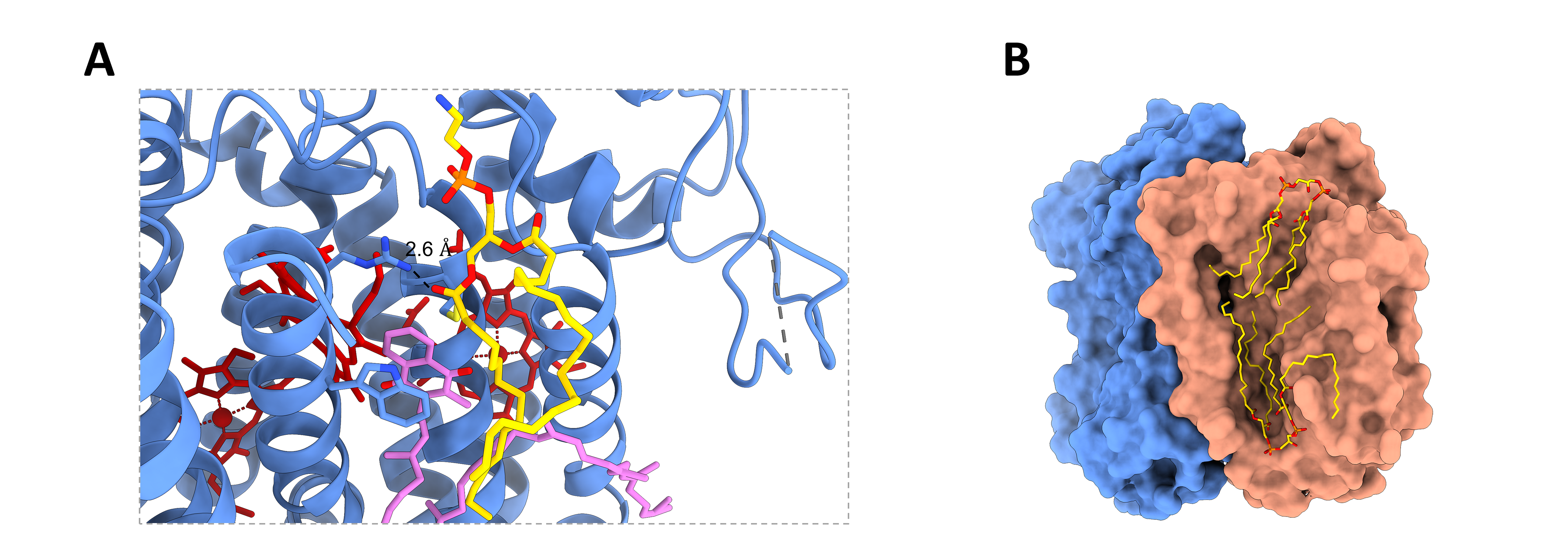


**SI Figure 6: Lipids resolved in the Mtbd structure. (A)** a PE lipid complementing the MK-9 binding pocket via hydrogen bonding to Arg8. **(B)** two cardiolipin molecules bound in the CydB hydrophobic groove.


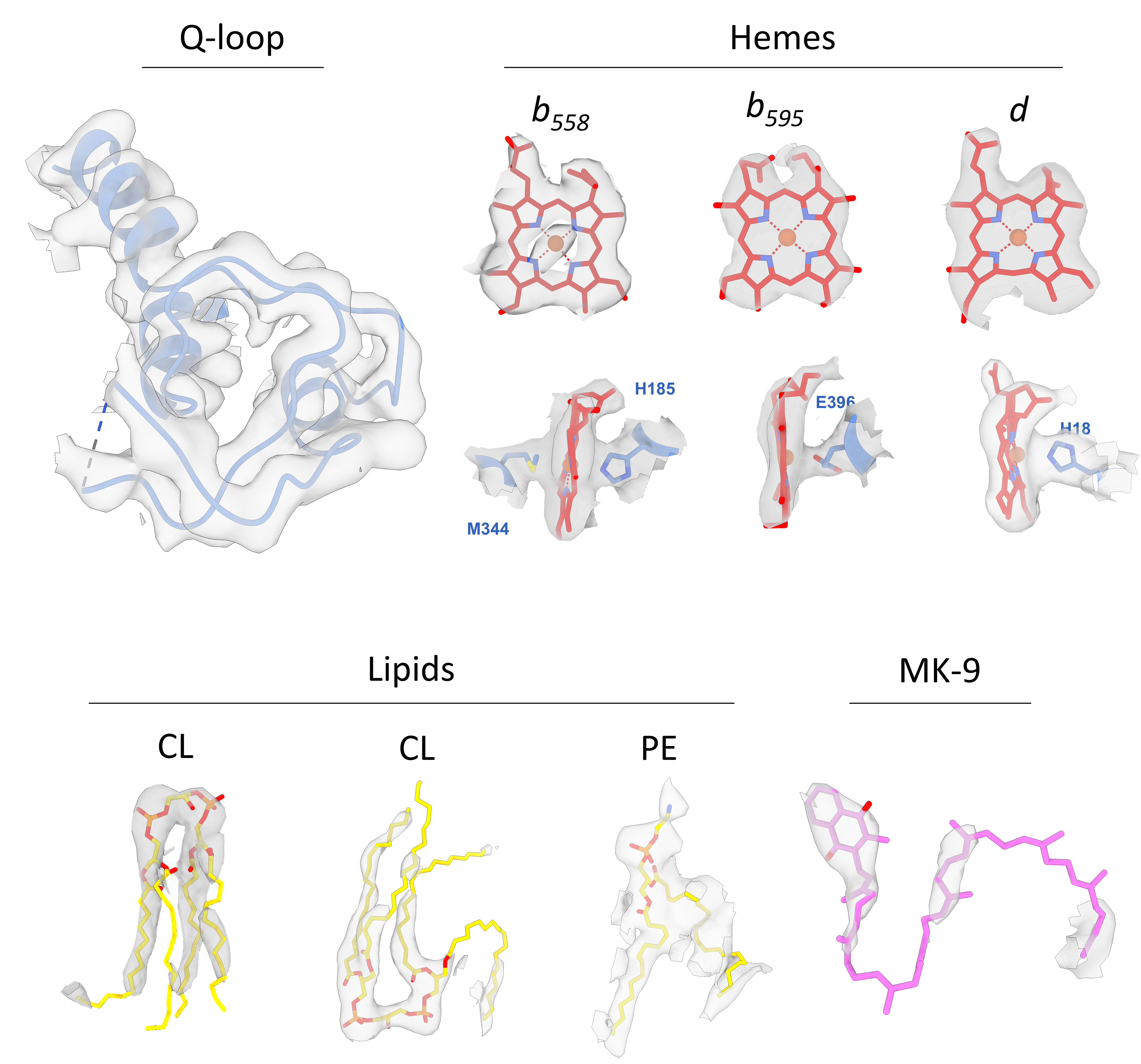


**SI Figure 7: Density features of the disulfide reduced Mtbd structure.**

**Supplementary references**

(1) Sharma, P.; Teixeira De Mattos, M. J.; Hellingwerf, K. J.; Bekker, M. On the Function of the Various Quinone Species in Escherichia Coli. In *FEBS Journal*; 2012; Vol. 279, pp 3364–3373. https://doi.org/10.1111/j.1742-4658.2012.08608.x.

(2) Unden, G.; Bongaerts, J. Alternative Respiratory Pathways of Escherichia Coli: Energetics and Transcriptional Regulation in Response to Electron Acceptors. *Biochim Biophys Acta* **1997**, *1320*, 217–234. https://doi.org/10.1016/s0005-2728(97)00034-0.

(3) Sajed, T.; Marcu, A.; Ramirez, M.; Pon, A.; Guo, A. C.; Knox, C.; Wilson, M.; Grant, J. R.; Djoumbou, Y.; Wishart, D. S. ECMDB 2.0: A Richer Resource for Understanding the Biochemistry of E. Coli. *Nucleic Acids Res* **2016**, *44* (D1), D495–D501. https://doi.org/10.1093/nar/gkv1060.
